# Translational research in agricultural biology—enhancing crop resistivity against environmental stress alongside nutritional quality

**DOI:** 10.3389/fchem.2014.00030

**Published:** 2014-06-05

**Authors:** Autar K. Mattoo

**Affiliations:** Sustainable Agricultural Systems Laboratory, United States Department of Agriculture, The Henry A. Wallace Beltsville Agricultural Research Center, Agricultural Research ServiceBeltsville, MD, USA

**Keywords:** antioxidants/nutrients, biotechnology, legume cover crops, genetic engineering, metabolomics, smart crops, sustainable agriculture, transcription factors

“Plants Are Smarter Than We Thought” was the headline news recently in a leading journal (Science News, March 6, 2014; http://news.sciencemag.org/signal-noise/2014/03/plants-are-smarter-we-thought), highlighting an article published elsewhere (Meyer et al., 2014), which presented evidence that plants are able to make smart decisions in response to predation and environment. Plants are well known to have evolved a fascinating adaptability to environment likely because of their sessile nature. Among a long list of complex and unique processes that plants have evolved include the oxygen evolving process of photosynthesis (Ort and Yocum, 1996; Demmig-Adams et al., 2006), which is the life force of animal/mammalian kingdoms, carried out by the semi-autonomous organelle, the chloroplast (Wise and Hoober, 2007); totipotency such that any cell from any plant part can divide, differentiate and yield a fully functional plant (Chupeau et al., 2013); the ability of and restoring structural (Meyer et al., 2014) and metabolic memory (Mattoo et al., 2007; Mattoo and Handa, 2008); the differentiated chromoplasts (from chloroplasts) that store important nutrients for animal and human health (Egea et al., 2010); long distance signaling up and down the whole plant (Ruiz-Medrano et al., 2001; Köhler and Mueller-Roeber, 2004); and recognition and communication via the emission of select class of volatiles (Holopainen and Blande, 2012; Das et al., 2013). Although the phenomenology is well described, the molecular and biochemical mechanisms involved in these processes are better known of some than other of these complex processes. The application of chemistry (and physics) principles has considerably added to the progress made in our understanding of plant life thus far.

Life on earth became possible some 3.5 billion years ago because “chemistry begat biology” (Aberlin, 2014). However, little is known or understood of what led to the transition from chemistry to biology (Quoted from Jack Szostak, Harvard Medical School, February 2012, as appeared in The Scientist 03-2014). Nonetheless, the past two centuries witnessed a close merger between chemistry (and physics) and biology, producing a distinct platform for biochemistry (Neuberg, 1903 in en.wikipedia.org/wiki/Carl_Neubergı) to bear on our understanding of the functions of a living cell and its complex nature. Because of its very nature, this discipline unearthed common and distinct alphabets and trends of biochemical processes that led to a concept of “unity in diversity,” exemplifying common principles that underlie the uniformity of life in diverse kingdoms. Kluyver's studies on microorganisms led to the discovery that ecological microbiology has a biochemical basis (Kluyver, 1924 in Florkin, 1924). The discovery that all the diverse organisms harbor same macromolecules and genetic code led to the knowledge that all organisms, from microorganisms to human beings, are built from similar molecular components with some variations (Berg et al., 2002).

The past century was a witness to advances in diverse disciplines including genetics, and micro- and macro-elements of biological chemistry. Thus, major discoveries were made on biochemical pathways (by Krebs, encompassing 1932, 1937, and 1957), cofactors (for instance, coenzyme A, by Fritz A. Lippman), enzymes (by Wilhelm Kuhne, as early as 1878), proteins (by Sumner), nucleic acids—DNA (by Watson and Crick) and RNA world (http://en.wikipedia.org/wiki/History_of_RNA_biology), cell membrane function and signaling pathways (http://en.wikipedia.org/wiki/History_of_biochemistry). These advances and discoveries brought together pieces of the puzzle(s) and laid the foundation for modern day molecular biology, biotechnology and epigenetic regulation. Progress in the identification and quantification of low abundant molecules led to Metabolome, which delves into the plasticity and/or homeostasis of primary and secondary metabolites, while small RNAs (including snoRNAs and miRNAs) brought to the fore the regulation by non-coding RNAs. Now and again, chemistry is having a bearing on the tremendous progress made in life sciences and our understanding of the biological processes.

The application of the innovative recombinant DNA technology enabled transfer of genes across kingdoms, creating the modern day biotechnological intervention to revolutionize research, and changing for good the paradigms in agricultural and medical research (Cohen et al., 1973). Transgenic research is the buzzword for all sorts of remedial measures, be that to prevent/solve diseases, produce recombinant products, or grow more food. Because of the totipotency of the plant cells, plant biologists were able to transform and develop plants engineered with novel, heterologous and endogenous genes. This has enabled unambiguous confirmation of gene function *in planta* and testing the novel genotypes for global changes in macromolecules and micromolecules alike. The detection, unambiguous identification, quantification and fast analysis of minutest amounts of cellular micromolecules including plant hormones has materialized through high resolution superior gas chromatography, ultra high performance liquid chromatography coupled to triple-quadrupole mass spectrometer, and Nuclear Magnetic Resonance (NMR) Spectroscopy, and this has become instrumental in understanding the chemical footprints during different phases of growth and development of plants.

The human genome sequencing was completed ahead of expected time, and this success catalyzed moves to sequence other genomes including crop plants, all expedited by the progress made in Information Technology - mining the data and putting pieces of the puzzle together in shortest time possible. Progress in the Genomics (http://www.sciencemag.org/site/feature/misc/webfeat/plantgenomes/feature.html) field ushered in the Epigenomics (Schmitz and Zhang, 2011) science, and together they have thrown the whole biological kingdom wide open to new research, furthering our understanding of the fundamental basis of life, development and regulation. The length and breadth of data that accumulate each day is humongous and has led to the active collaboration in the interface of bioinformatics and biology. This merging of two disciplines has enhanced the timely solutions and brought to bear on the way science in this century is being conducted worldwide.

More importantly, these advances in technology and biology have prepared the groundwork for the benefit of the humankind by providing reagents and roadmaps to solve the issues world is faced with at this time. This is most applicable and needed for human welfare, to have agricultural sustainability and feed the world, because there clearly is an increasing population growth trend throughout the globe except in Europe. World human population is expected to reach close to 9.6 billion by 2050 (http://esa.un.org/wpp/). The production capacity to grow more food to meet the demands of the burgeoning population gets complicated in view of the limits in the arable land that is less and less available, declining trends in crop yields, less sustainability of resources such as water, and losses due to abiotic and biotic stresses. Added to the challenge in producing more (nutritious) food is the need to fight malnutrition and reduce extensive chemical use in agriculture for a cleaner environment. Breeding strategies employing marker-assisted selection for high yielding varieties as well as for identifying germplasm resistant to abiotic and biotic stresses are already in vogue. Another approach is to introduce agronomically important genes and those that can help crops withstand environmental extremes into major and minor crops using genetic engineering technology.

## Making biotechnology more sustainable

Biological revolution—genetic engineering and biotechnology—has a promise to enhance crop resilience and make a breeder's dream come true: produce more in a shorter time, reduce our reliance on agricultural chemicals such as pesticides and fungicides, and add to environment-friendly sustainable agriculture (Mattoo, 2013). Some of the desirable traits that have been successfully introduced into crops by genetic engineering include insect resistance, disease resistance, herbicide tolerance, chilling tolerance, delayed fruit ripening, prolonged shelf-life, texture and processing attributes. Irrespective of this promise, the rapid pace in the development of novel engineered crops and the considerable interest generated among growers and consumers, the application of this technology and/or acceptance of genetically engineered foods world-wide has been hampered by continued debate on the safety of such produce. These issues include ethical concerns, potential toxicity, selection markers, undesired gene flow, development of resistance against herbicides and pesticides. Significant research efforts have gone into satisfying the consumers' concerns. Risk assessment studies have focused on determining the substantial equivalence of genetically engineered food and traditionally-bred wild type crops. In such studies, optimized unambiguous methodologies are required to search for differences between the engineered and non-engineered food. Many studies in the literature have not revealed any unusual compound in the genetically engineered crops, suggesting that they are basically substantially equivalent to non-engineered foods (Baker et al., 2006; Mattoo et al., 2006; Sobolev et al., 2010; Farre et al., 2011).

Plant-breeding programs normally include assessing and accounting the influence of genetic background (G), ecosystem environment (E), and G × E interactions singly and/or together on their impact on the growth and development of crops/plants, particularly for producing suitable genotypes for multiple environments (El-Soda et al., 2014). Evaluating these parameters also helps understand plant fitness trade-offs and evolutionary ecology El-Soda et al. (2014). Because the genetically engineered plants harbor a modified gene(s) and the positioning of the introduced gene is mostly a random event, it seems important that such plants be tested for agronomic and biological performance in the fields under multiple ecosystem services to assess which production system is conducive for impact on different parameters of the engineered plant(s) grown side by side the non-engineered wild type control(s). Studies of this kind/nature reported in the literature are miniscule.

It is imperative that controlled field studies of genetically engineered and other crops are carried out in an unambiguous manner alongside the wild type to ascertain which production system may provide the best medium. Such studies are expected to provide new ways to leverage growth enhancement, crop resistance to stresses, and improve the nutrient content of the edible produce in an eco-friendly environment. For instance, a major agriculturally utilized genetic event is the introduction of the *Bacillus thuringenesis* (Bt) protein gene in diverse crops, particularly cotton and maize, to make these crops resistant to insect pests (Marvier et al., 2007; James, 2013). The Bt crops have generated good revenue both for the farmer and the industry. However, like the non-engineered crops that suffer losses because of the adaptability of insects and microbial pathogens, it is expected that Bt crops and other such novel genetic materials will also be manipulated by the pathogens in the long run. Thus, in the field evaluation, populations of western rootworm were identified that had developed resistance to multiple Bt-maize toxins (Gassmann et al., 2014). In the above-mentioned context, i.e., developing a friendlier ecosystem suitable for growing each crop with a unique genetic event(s), it is of critical importance to understand their long-term performance in practical terms as well as a novel resource to discern various players in biological adaptability. Thus, studies that were geared to test the effect of natural predators in a defined ecosystem on the performance of Bt crops demonstrated that, indeed, natural enemies of insects help delay the development of insect resistance to Bt crops (Liu et al., 2014). Just as in conventional breeding strategies, so with novel biotech crops any unusual observation(s) in field performance behavior will have to be scientifically tackled and rectified.

## Developing robust crop plants to resist abiotic stressors

Plant growth in nature is compromised on a daily basis because plants expend energy to adjust and adapt to changing environment, which becomes more precarious under additional stresses due to drought and extreme temperatures, for instance, excessive summer temperatures of the tropics, or in cooler climatic situations. Thus, water availability, temperature, soil properties and ecosystem can dictate the growth response and yield of a crop plant. Moreover, each plant adapts to environment based on the genetic make up, accordingly impacting growth, development and yield (Porter and Semenov, 2005).

Molecular responses to unfavorable environment include a medley of genes and signal transduction pathways that are tightly regulated and empower plants to combat the stress conditions. Although much of this regulation is at transcriptional, post-transcriptional, and post-translational levels, the intricacy is of essence at the transcriptional level involving chromatin modification and remodeling, *cis*-regulatory elements located upstream and downstream the coding region of the gene, and *trans* regulatory transcription factors (Luo et al., 2012). Also, other important players that are directly or indirectly associated with imparting tolerance to abiotic stresses include protective proteins (including dehydrins, heat shock proteins—HSPs, Late Embryogenesis Abundant proteins—LEA Vierling, 1991; Wang et al., 2004; Kazuko and Shinozaki, 2006; Lipiec et al., 2013; Mu et al., 2013), osmolytes (proline/trehalose/sugars Fougere et al., 1991; Petrusa and Winicov, 1997; Wingler, 2002; Avonce et al., 2006; Ito et al., 2006; Ge et al., 2008; Zhang et al., 2010; Hayat et al., 2012; Yanhui et al., 2012), glycine betaine (Sakamoto and Murata, 2002; Quan et al., 2004; Wang et al., 2010; Chen and Murata, 2011), signaling molecules (polyamines Roy and Wu, 2002; Navakouidis et al., 2003; Capell et al., 2004; Kasukabe et al., 2006; Alcázar et al., 2010; Wi et al., 2006; Liu et al., 2007; Kusano et al., 2008; Wen et al., 2008; Cheng et al., 2009; Kalamaki et al., 2009; Gill and Tuteja, 2010; Shukla and Mattoo, 2010; inositol Xiong et al., 2001; Sengupta et al., 2008); and hormones (abscisic acid—ABA Davies and Zhang, 1991; Saradhi et al., 2000; ethylene—C_2_H_4_ Hinz et al., 2010; Quan et al., 2010; Xiong et al., 2013; and methyl jasmonate—meJA Bartels and Sunkar, 2005; Vincour and Altman, 2005; Wu et al., 2008; Jan et al., 2013), several of which have been validated for mitigating abiotic stresses.

**Figure 1:**
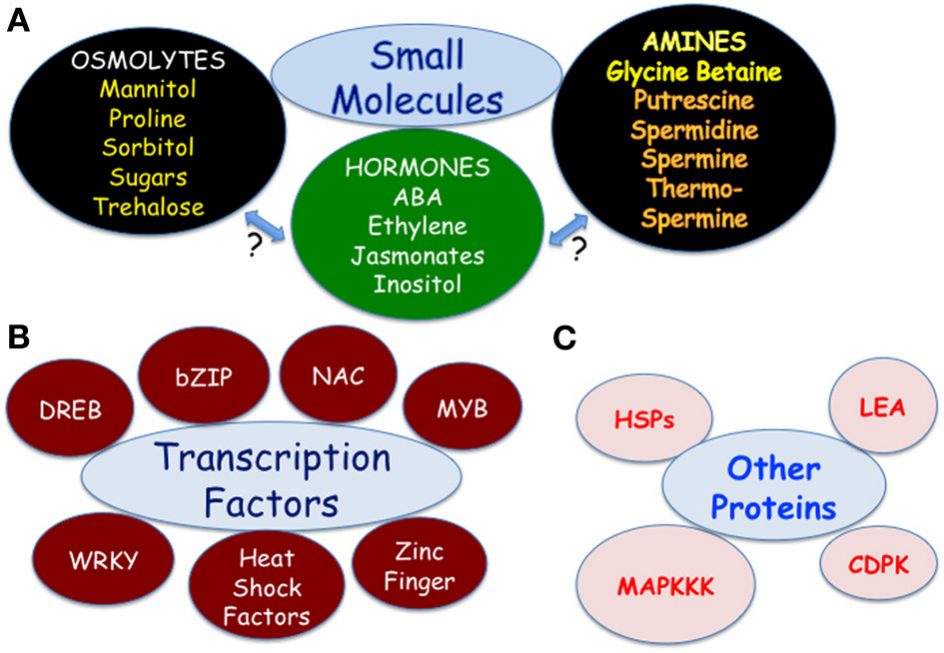
**Players that empower crop plants to withstand abiotic stresses—drought, temperature extremes and high saline soils. (A)** Small molecules such as osmolytes, biogenic amines and hormones. **(B)** Transcriptional factors. **(C)** Heat shock proteins (chaperone proteins) and protein kinases.

Genome sequencing of model and crop plants before and after exposure to a given stress has identified candidate genes whose role(s) in response to different abiotic stresses can then be tested/validated by expression and down-regulation in homologous as well as in heterologous systems. Thus, stress responsive genes including specific transcription factors have been identified by comparative transcriptomics. Enormous activity regarding validated data on a few crops for the involvement of transcription factors (b-ZIP, ERF/AP2 family, DOF, HD-ZIP, MYB, NAC, WRKY, and Zn-finger) (Riechmann and Ratcliffe, 2000; Dubouzet et al., 2003; Hu et al., 2006; Ito et al., 2006; Mittler, 2006; Nakashima et al., 2007; Weiste et al., 2007; Wu et al., 2008; Xiang et al., 2008; Zou et al., 2008; Gao et al., 2009; Lu et al., 2009; Oh et al., 2009; Jeong et al., 2010; Su et al., 2010; Takasaki et al., 2010; Zhang et al., 2010; Zhao et al., 2010; Wan et al., 2011; Liu et al., 2012; Yang et al., 2012; Jan et al., 2013), and other genes (CDPKs, HAP/CAAT, HSPs-LEA family, MAPKKK) (Vierling, 1991; Saijo et al., 2000; Wang et al., 2004; Chandra Babu et al., 2004; Shou et al., 2004; Kazuko and Shinozaki, 2006; Xu et al., 1996; Nelson et al., 2007; Xiao et al., 2007; Ning et al., 2010; Duan and Cai, 2012; Lipiec et al., 2013; Mu et al., 2013) have shown the true promise of these candidates as stress modulators. Members from each transcription factor family show protective phenotypes against multiple stresses such as cold, drought and excess salt (summarized in Figure 1; Shukla and Mattoo, 2013; Mattoo et al., 2014). Similarly, engineering targeted metabolic pathways enable multi-throng efforts to produce and sustain agricultural commodities for the benefit of the farmer and the consumer (Reugera et al., 2012).

Such successful efforts on engineering crop plants for resistance to different abiotic stresses have started paying dividends since industry has generated some drought resistant germplasm for the farmers. This progress in translating basic research into viable products offers a roadmap for intensifying efforts to develop resistant germplasm for all the major and minor crops and test them under varied climatic conditions and different ecosystems worldwide.

## Nutrient-enhanced produce and human health

It is more and more recognized that phytonutrient-rich diet containing high dose of antioxidants/vitamins, present in fruit and vegetables, potentially prevents polygenic diseases such as epithelial cancers, diabetes, atherosclerosis, hypertension, cardiovascular diseases and osteoporosis (Mattoo et al., 2010; Shukla and Mattoo, 2010; Fatima et al., 2013). This is the reason that antioxidant/vitamin supplements are available over the counter in pharmacies and grocery stores worldwide. The well-known health-promoting phytonutrients that have been proposed to alleviate disease symptoms and reduce incidence of diseases include carotenoids (β-carotene, lutein, lycopene), polyphenolics/flavonoids, vitamins C and E, isothiocyanates and glucosinolates. That disease and nutrition are intertwined in humans is becoming relevant in this science/technology, post-genomic era and the beneficial effects seem linked to interactions among different antioxidants present in food, although little is known about the nature of these interactions (Shukla and Mattoo, 2010; Fatima et al., 2013). Thus, the need for a dense nutrient intake through eating a variety of foods including grains, fruits and vegetables has been emphasized in the dietary guidelines for Americans (http://www.dietaryguidelines.gov).

The increasing interest in the bioactive molecules present in grains, fruits and vegetables has catalyzed research interest in developing definitive basic information on their content, with more than 40 such molecules having been identified and deemed essential for a healthy life (Failla, 2012). The quantity required of each nutrient to decrease “disease risk” is a critical factor while bioavailability of nutrients in a diet determines how much of the good nutrient's potential is realized (Shukla and Mattoo, 2010; Fatima et al., 2013). Although grains, vegetables, and fruits are sources of antioxidants and vitamin nutrients, the levels in general are low, likely due to tight genetic and developmental controls of their metabolic pathways during plant growth and development (Paine et al., 2005). Further, biosynthetic pathways for phytonutrients and their regulation are incomplete in many instances, and “germplasm” with higher accumulation of phytonutrients is not easily available. Modern tools of metabolomics have already overcome these limitations by identifying, purifying and quantifying hundreds of biochemicals (Mattoo et al., 2006; Saito and Matsuda, 2010). Mutagenesis and TILLING (Targeting Induced Local Lesions in Genomes) approaches are being used to use selection against genes that negatively regulate biosynthesis or accumulation of phytonutrients (Zhang et al., 2009; Handa et al., 2011).

Molecular genetics is now providing tools to identify and characterize genes regulating the biosynthesis of phytonutrients in plants. Thus, genetic/metabolic engineering of the rate limiting steps in the biosynthesis of a compound has facilitated increased levels of phytonutrients in plant tissues/organs (Shukla and Mattoo, 2010; Fitzpatrick et al., 2012; Fatima et al., 2013; Handa et al., 2014). Animal and human trials can help determine which nutrient(s) needs to be enhanced through molecular strategies designed to increase their contents in grains, vegetables, and fruits. Interestingly, the human health paradigm has been revised to include preventive, dietary intervention to ameliorate diseases and physiological disorders. More scientific research and validation are required before phytonutrients become a “mantra” for healthy living (Handa et al., 2014).

Regulatable promoters fused to heterologous genes allowed higher levels of carotenoids to accumulate in a fruit-specific manner in tomato (Rosati et al., 2000; Dharmapuri et al., 2002; Fraser et al., 2002; Mehta et al., 2002), and similarly in other instances where constitutive promoters were employed (Römer et al., 2000; D'Ambrosio et al., 2004). Use of regulatable promoters fused to the *E. coli DXS* gene (Enfissi et al., 2005) or suppression RNAi to downregulate the photomorphogenesis regulatory protein gene *DET1* (Davuluri et al., 2005) also led to high levels of carotenoids in tomato fruit. Also, metabolic engineering of polyamine biosynthesis in tomato by introducing fruit-specific expression of yeast S-adenosylmethionine (SAM) decarboxylase gene led to 200–300% increase in lycopene content (Mehta et al., 2002) while constitutive expression of yeast spermidine synthase increased carotenoid content by 40% (Nambessan et al., 2010). *TOMATO AGAMOUS-LIKE 1* (*TAGL1*), a *MADS*-box transcription factor, expression resulted in higher accumulation of lycopene and naringenin chalcone (Itkin et al., 2009). Another study used RNAi-mediated fruit-specific suppression of 9-cis-epoxycarotenoid dioxygenase 3 (*NCED3*) to suppress ABA synthesis to stimulate accumulation of upstream compounds such as β-carotene and lycopene in transgenic tomato fruits (Sun et al., 2012). In this regard, a mutation in *zep1* caused ABA-deficiency in tomato plants with concomitant accumulation of 30% more carotenoids in mature red tomato fruit (Galpaz et al., 2008).

A few other selective examples of engineering phytonutrient content are:

GDP-L-galactose phosphorylase (*VTC2*) expression increased vitamin C level in tomato, strawberry, and potato by 2-6 fold (Bulley et al., 2012); mammalian *GTP cyclohydrolase I* caused 140-fold increase in pteridine and 2-fold increase in folate, and by combining with aminodeoxychorismate synthase (PABA biosynthesis), folate levels were increased 19-fold (Diaz de la Garza et al., 2007); expression of α-tocopherol methyltransferase and γ-tocopherol methyltransferase, respectively in soy oil and lettuce, increased vitamin E several-fold (see Fatima et al., 2013); simultaneous expression of genes for β-carotene, ascorbate, and folate biosynthetic pathways increased β-carotene (169-fold), ascorbate (6-fold), and folate (2-fold) in corn (Naqvi et al., 2009); expression of *Rosea1* and *Delila* or flavonoid-related *R2R3-MYB* increased flavonoid content in tomato pericarp (Butelli et al., 2008); RNAi suppression of the *DE-ETIOLATED1* (*DET1*) gene (a photomorphogenesis regulatory gene) caused several-fold increase in carotenoid, tocopherol, phenylpropanoids and flavonoids (Enfissi et al., 2010); metabolic engineering of diverse genes that encode enzymes for secondary metabolites all increased content of polyphenolic flavonoids in tomato fruit (Muir et al., 2001; Niggeweg et al., 2004; Giliberto et al., 2005; Schijlen et al., 2006), α-tocopherol (vitamin E) in potato tubers (Crowell et al., 2008), and some produced novel flavonoids in tomato fruit (Schijlen et al., 2006).

It is apparent that modern biotechnology in conjunction with Metabolomics is enabling tissue specific redesign of primary and secondary metabolic pathways so as to accumulate high levels of phytonutrients in different plant systems. Thus, transgenic crops are an addition to the genetic resource to further define genetic, biochemical, and physiological regulation of cellular metabolism pathways, including enhancing functional metabolites in produce and provide novel “specialty crops” to the public. In return, public awareness of benefits of consumer-driven products such as in human health will further add to new markets for specialty, highly nutritious crops.

## Future perspective

Agricultural biologists have their work cut out for translating large database of fundamental nature from laboratory, growth chamber and greenhouses studies to the field for securing and producing (nutritious) food and making agriculture sustainable. All kinds of transgenic lines have been developed, including transgenic lines that have promise of withstanding environmental extremes (abiotic and biotic) and others that have a high dose of phytonutrients. How they fare in the field is the need of the day, effectiveness of this translation will require diligence and a thorough knowledge of the investigated trait in each crop (Ronald, 2011; Nelissen et al., 2014). Moreover, there appears to be a probability that ecological surprises could be more prevalent because of global climate change and interacting environment extremes (Lindenmayer et al., 2010). Also, the point to note is that nutrient levels in crops are influenced by genotype/cultivar, growth condition and developmental stage of the crop (Shukla and Mattoo, 2010; Lee and Scagel, 2014), therefore unambiguous analysis of edible crops grown under similar conditions in the field is needed to determine the robustness of a trait (Neelam et al., 2008; Mattoo and Teasdale, 2010). Convergence of agriculture with health and wealth is a distinct possibility (Dube et al., 2012), and would also be benefited by developing necessary toolkits to establish *bon a fide* natural products chemistry and translate it into alternative medicine.

Thus, with the available genetic toolkits together with advanced technologies, chemical genetics, and progression with alternative agricultural practices, future action plan is more or less laid out and roadmap defined for scientists and farmers to work together to meet the challenges the humankind faces in this new century. It is evident that there is a need to prioritize translational research as an important component of bench scientists' goals of research. Certainly, there is hope in the horizon for developing new types of crop plants that can yield more and be nutritious with less inputs, are resilient to harsher environment, and are disease tolerant.

### Conflict of interest statement

The authors declare that the research was conducted in the absence of any commercial or financial relationships that could be construed as a potential conflict of interest.
